# Histone modifications as regulators of life and death in *Saccharomyces cerevisiae*

**DOI:** 10.15698/mic2016.01.472

**Published:** 2015-12-31

**Authors:** Birthe Fahrenkrog

**Affiliations:** 1 Institute of Molecular Biology and Medicine, Université Libre de Bruxelles, Rue Profs. Jeener et Brachet 12; 6041 Charleroi, Belgium.

**Keywords:** apoptosis, autophagy, epigenetics, histone modification, Saccharomyces cerevisiae, yeast

## Abstract

Apoptosis or programmed cell death is an integrated, genetically controlled suicide program that not only regulates tissue homeostasis of multicellular organisms, but also the fate of damaged and aged cells of lower eukaryotes, such as the yeast *Saccharomyces cerevisiae*. Recent years have revealed key apoptosis regulatory proteins in yeast that play similar roles in mammalian cells. Apoptosis is a process largely defined by characteristic structural rearrangements in the dying cell that include chromatin condensation and DNA fragmentation. The mechanism by which chromosomes restructure during apoptosis is still poorly understood, but it is becoming increasingly clear that altered epigenetic histone modifications are fundamental parameters that influence the chromatin state and the nuclear rearrangements within apoptotic cells. The present review will highlight recent work on the epigenetic regulation of programmed cell death in budding yeast.

## INTRODUCTION

Apoptosis or "programmed cell death" is a self-destructing process important for the development and homeostasis of multicellular organisms and its deregulation contributes to the pathogenesis of multiple diseases including autoimmune, neoplastic and neurodegenerative disorders 1. Apoptosis is characterized by biochemical and morphological rearrangements throughout the cell 2 with chromatin condensation conjoint by DNA fragmentation being one of the most important nuclear alterations 3. The mechanism by which chromosomes reorganize during apoptosis is still poorly understood, but recent years have shown that epigenetic changes of the chromatin state are fundamental parameters of the nuclear rearrangements experienced by apoptotic cells. 

Chromatin is a composite of packaged DNA and associated proteins, in particular histones 4. The basic subunit of chromatin is the nucleosome containing 147 base pairs of DNA, which are wrapped around a histone octamer containing two copies each of the core histones H2A, H2B, H3 and H4. Nucleosomes are then packaged into higher order structures in a yet controversially discussed manner 5, in which individual nucleosomes are separated from each other by the linker histone H1 and its isoforms. The tails of the core histones pass through channels within the DNA molecule away from it and are subjected to a wide variety of post-translational modifications. These post-translational modifications include lysine acetylation, butyrylation, propionylation, ubiquitination and sumoylation, lysine and arginine methylation, arginine citrullination, serine, threonine and tyrosine phosphorylation, proline isomerization as well as ADP ribosylation 6,7,8,9,10. In the context of apoptosis in particular phosphorylation and acetylation of histones have long been suggested to affect chromatin function and structure during cell death 11. 

The budding yeast *Saccharomyces cerevisiae* has matured as attractive model system for apoptotic research to study the evolutionary conserved aspects of programmed cell death. Apoptosis in* S. cerevisiae* can be activated by various agents including hydrogen peroxide (H_2_O_2_), acetic acid and pheromone or by physiological triggers, such as DNA replication stress, defects in DNA damage repair, chronological or replicative aging and failed mating 12,13,14,15,16. The chronological lifespan (CLS) is defined as the time yeast cells remain alive in a post-mitotic, quiescence-like state 17,18,19 and genetically, amongst others, strongly influenced by key apoptotic regulators, such as Aif1, Bir1, Nma111, Nuc1, Ybh3 and Yca1 20,21,22,23,24,25,26,27,28, all of which have homologs in mammals. Replicative aging, the second type of aging studied in yeast, is defined by the number of cell divisions an individual mother cell can undergo before entering senescence (replicative lifespan, RLS) and is equally controlled by some of these genetic factors as well as by environmental conditions 17,19. Chronological and replicative aging both lead to an accumulation of reactive oxygen species (ROS) that ultimately results in programmed death of the budding yeast cells 24,29. 

As in higher eukaryotes, it is becoming more and more evident that the programmed death of *S. cerevisiae* is largely influenced by epigenetic modifications, in particular phosphorylation of H2B and H3, acetylation of H3 and H4, deubiquitination of H2B, as well as methylation of H3 30,31,32,33,34,35. This review will highlight current knowledge on the posttranslational histone modifications that decide on yeast life and death.

## EPIGENETIC REGULATION OF YEAST LIFE AND DEATH

### Histone phosphorylation

In metazoans, the first histone modification that had been linked to apoptosis was phosphorylation of the histone variant H2A.X at serine 139 (S139), known as γ-H2AX, that occurs during the formation of DNA double strand breaks (DSBs) under various conditions, including apoptosis 36. Furthermore, phosphorylation of histone H2B at S14 had been associated with chromatin condensation and DNA fragmentation 37,38,39. H2B phosphorylation is reciprocal and deacetylation of H2B at lysine 15 (K15) is necessary to allow H2BS14 phosphorylation 40. 

**Figure 1:**
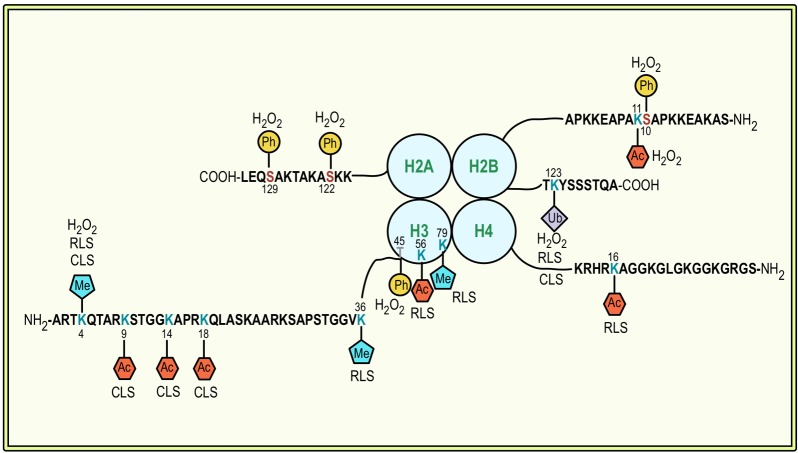
FIGURE 1: Specific histone modifications that have been shown to be associated with apoptotic cell death and lifespan regulation in *S. cerevisiae*. Modified lysine residues are highlighted in cyan, modified serine residues in red, and modified tyrosine residues in grey. Ac, acetylation; Me, methylation; Ph, phosphorylation; Ub, ubiquitination.

This unidirectional crosstalk between two histone modifications was originally revealed in yeast: phosphorylation of S10 of H2B (H2BS10ph; Fig. 1) is essential for the induction of an apoptotic-like cell death in H_2_O_2_-treated cells 30. H2BS10A point mutants exhibit increased cell survival accompanied by a loss of DNA fragmentation and chromatin condensation, whereas H2BS10E point mutants display the typical phenotypic markers of apoptosis, including chromatin compaction and DNA fragmentation. Triggering apoptosis by H_2_O_2_, acetic acid or the α-factor leads to phosphorylation of H2BS10 and H2BS10ph is preceded by H2A phosphorylation and mediated by the Sterile 20 kinase, Ste20 (Table 1) 30. It is dependent on the yeast metacaspase Yca1 and the preceding deacetylation of lysine 11 31. 

**Table 1:** Histone modifications involved in aging and apoptotic processes in yeast. The implications listed are referring to the respective histone modifications. The listed writers and erasers may have impact on other histone modifications not related to cell death as well and the modifiers may have targets other than the histones, which may implicate them in other cell death pathways.

**Histone**	**Modification**	**Writer**	**Eraser**	**Implication**	**Reference**
H2A	S122 phosphorylation			H_2_0_2_	48
H2A	S129 phosphorylation	Mec1, Tel1	PPH3	H_2_0_2_	152,153
H2B	S10 phosphorylation	Ste20		H_2_0_2_	30
H2B	K11 acetylation		Hos3	H_2_0_2_	31
H2B	K123 ubiquitination	Rad6/Bre1	Ubp10, Ubp8	CLS, RLS, H_2_0_2_	32,35,49,50,53,72,73
H3	K4 methylation	Set1/COMPASS	Jhd2	CLS, RLS, H_2_0_2_	33,35,57,121,154,155
H3	K36 methylation	Set2	Jhd1	RLS	124,143,156
H3	K79 methylation	Dot1		RLS	33,129
H3	T45 phosphorylation	AKT1, 2		H_2_0_2_	47,157
H3	K9 acetylation	Gcn5		CLS	161,164
H3	K14 acetylation	Sas3	Hos3	CLS	161,164
H3	K18 acetylation	Gcn5		CLS	161,164
H3	K56 acetylation	Asf1, Rtt109	Hst3, Hst4	RLS	91,152,153,158
H4	K16 acetylation	Sas2	Sir2	RLS	87,159,160

H2B lysine 11 is acetylated (H2BK11ac) in logarithmically growing yeast 41 and deacetylated upon H_2_O_2_ treatment before H2BS10ph occurs. H2BK11ac was found to be present through 60 min of H_2_O_2_ treatment and the disappearance of K11ac after 90 min post H_2_O_2_-induction coincided with the onset of S10ph in H2B 31. The crosstalk between H2BS10ph and H2BK11ac is unidirectional and confirmed by H2BK11 mutants: lysine-to-glutamine H2B K11Q mutants, that are acetyl-mimic, are resistant to cell death elicited by H_2_O_2_, while lysine-to-arginine H2B K11R mutants that imitate deacetylation promote cell death. Deacetylation of K11 is mediated by the histone deacetylase (HDAC) Hos3 (Table 1)^31^. 

Interestingly, in human cells it has been shown that H2BS14ph, which is mediated by caspase-activated kinase Mst1, is read by RCC1 42. RCC1 is chromatin-bound and the guanine nucleotide exchange factor for the RanGTPase, which acts as a molecular switch to regulate directionality of nucleocytoplasmic transport as well as distinct steps of mitosis, such as spindle and nuclear envelope assembly 43,44,45. H2BS14ph immobilizes RCC1 on chromatin, which causes a reduction of nuclear RanGTP levels and the inactivation of the nucleocytoplasmic transport machinery 42, which in turn contributes to the inactivation of survival pathways, such as NF-κB signaling 11. Whether H2BS10ph in yeast has a similar effect on the nucleocytoplasmic transport machinery and survival pathways remains to be studied.

A potential trans-histone crosstalk related to yeast apoptosis occurs between H2A and H3 phosphorylation: phosphorylation of H2AS129, which resembles γ-H2AX of higher eukaryotes 46, is increasing in yeast cells undergoing H_2_O_2_-induced apoptosis and it is paralleled by a decrease in phosphorylation of threonine 45 in histone H3 (H3T45ph) 47. On the other hand, the function of H3T45ph in apoptotic signalling has been questioned and rather been linked to DNA replication and its absence with replicative defects 11. Oxidative damage of DNA by menadione, a reactive quinone that as H_2_O_2 _generates ROS, has furthermore been shown to lead to phosphorylation of H2A at serine 122 (H2AS122), and serine-to-alanine H2AS122A mutants have impaired survival on plates containing DSB-inducing drugs, such as methyl methanesulfonate (MMS) or bleomycin 48, supporting the potential importance of H2A phosphorylation in apoptotic signalling during DNA damage response. However, further investigations are necessary to provide mechanistic insights.

### Histone H2B ubiquitination

Another apoptosis-related modification of H2B is its ubiquitination (Fig. 1). H2B is monoubiquitinated (H2Bub1) at K123, which, in *S. cerevisiae*, is mediated by the macromolecular complex containing the E2-conjugating enzyme Rad6 and the E3 ligase Bre1 (Table 1) 49,50,51. H2Bub1 has been linked to transcriptional activation and elongation and *BRE1* disruption or lysine-to-arginine substitution at K123 of H2B (H2B-K123R) results in a complex phenotype that includes failures in gene activation 52,53,54,55 and lack of telomeric silencing 56,57,58,59. H2Bub1 is important for nucleosome stability 60, it marks exon-intron structure in budding yeast 61 and prevents heterochromatin spreading 62, and it is implicated in DNA repair and checkpoint activation after DNA damage 63,64 and during meiosis 65. The function of H2Bub1 and Bre1 and its homologues in transcription regulation and DNA damage response appears conserved across evolution 66,67,68,69,70. In human cells and in *Drosophila* it was furthermore shown that H2Bub1 is facilitated by O-linked N-acetylglucosamine modification of H2B at S112 71.

The DNA damage response machinery is closely linked to apoptosis in yeast and higher eukaryotes 14. The observation that a loss of the ubiquitin-specific protease* UBP10,* which is involved in cleaving the ubiquitin moiety from H2B (Table 1) 72,73, activates the yeast metacaspase Yca1 and apoptosis 74, were first hints that H2B ubiquitination may in fact be involved in the regulation of yeast apoptosis. The finding that enhanced expression of Bre1 protected yeast cells from H_2_O_2_-induced cell death, whereas deletion of *BRE1* potentiated cell death verified this notion 32. 

During chronological aging, cells lacking *bre1* show shortened lifespan that coincides with the appearance of typical apoptotic markers, such as DNA fragmentation and accumulation of ROS. The ability of Bre1 to reduce cell death is conferred by its E3 ubiquitin ligase activity mediated by its C-terminal zinc-binding RING finger domain 50. RING domains are frequently found in E3 ubiquitin ligases and required for catalysing the transfer of ubiquitin from the E2 to the substrate 75. ∆*bre1* cells complemented with a RING finger mutant of Bre1 (C648G, C651G) lack H2Bub1 and exhibited increased apoptosis sensitivity similar to ∆*bre1* cells, whereas the complementation of ∆*bre1* cells with a functional Bre1 made the cells behave like wild-type 32. Furthermore, H2B-K123R mutant cells, which too lack H2Bub1, have an increased sensitivity to apoptotic stimuli, exactly as ∆*bre1* cells. 

Yeast cells deficient for *ubp10* display markers of apoptosis, such as DNA fragmentation, as well as enhanced expression of stress-responsive genes as compared to wild type 76. The increased sensitivity to apoptosis observed in both ∆*bre1* and ∆*ubp10* strains is associated with an increase in the activity of Yca1, while deletion of *yca1 *restored the wild-type phenotype 32,74. Deletion of Silencing Information Regulator 2 (Sir2), a NAD^+^-dependent HDAC that amongst a plethora of targets predominately removes acetyl groups from K16 of histone H4 (Table 1) 77, could partially rescue the transcriptional pattern and abrogate the apoptotic effects of ∆*ubp*10 cells, suggesting that increased *YCA1* expression may result from inappropriate localization of silencing complexes upon failed deubiquitination of H2B 72,73,76.

H2B ubiquitination is also an important regulator of the replicative lifespan of *S. cerevisiae*: replicatively aged cells have increased H2Bub1 in their telomeric heterochromatin, along with increased methylation of histone H3 at lysine 4 and 79 (H3K4 and H3K79; see section "Histone methylation"), respectively 35,78. Yeast aging is accompanied by the loss of transcriptional silencing at the three heterochromatic regions of the yeast genome: at least at one telomere 79, at the mating type locus 80 and of rDNA 81. A key regulator of telomeric heterochromatin and rDNA silencing is Sir2 82,83,84,85,86, which is furthermore of vital importance for RLS regulation: its over-expression extends lifespan 87, while yeast cells lacking *sir2* have a shorter lifespan 88. The effect on the RLS is likely due to Sir2's ability to repress rDNA recombination 89, which in turn hampers the formation of extrachromosomal rDNA circles 87. The protein levels of Sir2 typically decrease during aging, which leads to increased levels of acetylated histone H4 at lysine 16 (Fig. 1) and a concomitant loss of histones from specific subtelomeric regions of the genome 90. The increase in H2Bub1 in replicatively aged yeast cells coincides with decreased Sir2 abundance and an increase in acetylation of histone H4 at K16 (H4K16ac; see section "Histone acetylation") along with much lower occupancies of H3, H4, or H2B at the heterochromatic regions 35. A global loss of histones during aging was previously also observed by Feser *et al.* 91. In addition to H2Bub1, also methylation of H3K4 and H3K79 are enriched in aged cells at regions proximal to telomeres 35. Consequently deficiencies in *rad6* and *bre1* and the expression of H2B-K123R cells reduce the mean lifespans of yeast cells, which is not further reduced by deletion of *sir2*. Together these data indicate that H2B monoubiquitination and methylation of H3K4 and K3K79 regulate replicative aging through a Sir2-related pathway 35. 

Regulation of apoptosis by H2Bub1 may further exist in multicellular organisms: deletion of Rfp1, the Bre1 homologue in *Caenorhabditis elegans*, leads to enhanced germ cell apoptosis in the worms 92 and the depletion of Bre1b, one of the two Bre1 isoforms in mice, leads to a strong increase in apoptosis frequency in different mouse cell types 93. In higher eukaryotic cells, however, ubiquitinated H2A is the most abundant species 94,95,96,97 and might assume some of the roles ubiquitinated H2B plays in yeast. Therefore H2A deubiquitination has been linked to chromatin condensation in mitotic and apoptotic cells in higher eukaryotes and the disappearance of H2Aub1 to late apoptotic events 98. In fact, rapid and extensive deubiquitination of H2A occurs in Jurkat cells undergoing apoptosis initiated by, for example, anti-Fas activating antibody, staurosporine, etoposide, doxorubicin and proteasome inhibitors 99, indicating that histone deubiquitination, either H2A or H2B, is a common apoptotic trigger across evolution.

### Histone methylation

Monoubiquitination of H2B is prerequisite to the di- and tri-methylation of histone H3 on lysine 4 (H3K4me2/3) and lysine 79 (H3K79me2/3) 59,100,101. This crosstalk is unidirectional, as mutations that eliminate either H3 modification had no effects on the level of H2B ubiquitination 100. H3K4 trimethylation is further regulated by monoubiquitination-independent processes: loss of H3K14 acetylation results in the specific loss of H3K4me3, but not mono- or dimethylation 102 and methylation of H3 at arginine 2 (H3R2) disables H3K4 methylation in yeast and mammalian cells 103,104,105. H3K4me3 marks localize to the 5’ end of active genes in budding yeast and are found associated with the initiated, phosphorylated form of RNA polymerase II 106,107,108, implicating it in transcriptional elongation. It is further not only important for transcriptional activation 109,110,111,112, but also for silencing at telomeres 112,113,114 and rDNA loci 57,115,116. Yeast cells lacking *set1* are deficient in the non-homologous end joining pathway of DSB repair and are impaired in traversing S-phase of the cell cycle in the presence of replication stress 117. In fact, *set1* deficient cells have a reduced replication activity 118. H3K4 methylation is mediated by the Set1-containing complex COMPASS (Table 1), which, in *S. cerevisiae*, consists of seven subunits 119,120,121,122,123 and is highly conserved among eukaryotes 61. With respect to apoptosis it has been shown that a loss of H3K4me3 (Fig. 1) is a trigger for apoptotic cell death 33. Strains lacking *set1* (or two other members of the COMPASS complex, Spp1 and Bre2, respectively) are susceptible to Yca1-dependent apoptosis, both during chronological aging as well as in response to H_2_O_2_ treatment 33. ∆*set1* cells similarly have a shortened RLS 124. Preventing loss of H3K4me3 by depleting the H3K4 demethylase Jhd2 on the contrary prolonged the CLS of the cells 33.

H3K79me3 is mediated by the methyltransferase Dot1 (Disruptor Of Telomeric silencing 1; Table 1). Dot1 is highly conserved across evolution and appears to be the sole methyltransferase responsible for H3K79 methylation 125,126,127,128,129,130. H3K79 methylation is essential for efficient silencing near telomeres, rDNA loci, and the yeast mating type loci 113, for precise DDR 63,131,132,133,134, and in higher eukaryotes for transcriptional control of developmental genes, such as *HOXA9* 108,135 and Wnt target genes 136. Contrary to H2Bub1 and H3K4me3, H3K79me3, however, appears to play no primary role in apoptotic signalling in yeast: deletion of *dot1* only slightly improves survival of wild-type cells, but it rescues ∆*set1* cells from apoptotic death 33. Furthermore, the DNA damage checkpoint kinase Rad9 and the yeast homolog of endonuclease G, Nuc1, are critical for cell death of ∆*set1 *cells, suggesting that loss of H3K4 methylation in the presence of H3K79 methylation and the kinase Rad9 enhances chromatin accessibility to endonuclease digestion. Importantly, wild-type, but not *dot1*∆ cells, loose H3K4 methylation during chronological aging, which coincides with a shorter lifespan and indicates that the loss of H3K4 methylation in fact acts as important trigger for apoptotic cell death 33. 

A link between apoptosis and H3K4me was also observed in other species, although data here are somewhat controversial: while deficiency in MLL1, one of several H3K4 methyltransferases in mammals 137, and a lack of H3K4me3 was enhancing apoptosis induced by ER-stress 138, H3K4me3 recognition was necessary to stimulate DNA-repair after UV irradiation and to promote DNA-damage- 139,140 or genotoxic stress-induced apoptosis 141, indicating that regulation of apoptosis by H3K4 methylation may occur in a context- and/or tissue-specific manner.

As outlined above, replicatively aged cells have increased H2Bub1, H3K4me, and H3K79me in their telomeric heterochromatin, which is accompanied by the loss of transcriptional silencing at the telomeres. The anti-silencing activity of H2Bub, H3K4me, and H3K79me at telomeric regions is opposed by another histone methyltransferase: Set2 35,124. Set2 mediates methylation of H3 at K36 (Table 1) 142,143, which occurs independent of H2Bub1 100,144. Methylated H3K36 has been implicated in transcriptional elongation and H3K36me3 is typically found to accumulate at the 3’end of active genes in association with the phosphorylated elongating form of RNA polymerase II 142,145,146,147. The anti-silencing function of Set2 was observed at all three heterochromatic regions in yeast and *set2*-depleted cells have a prolonged RLS as compared to wild-type cells 35,124. Contrary to yeast, loss of H3K36me3 and deletion of the mediating methyltransferase *met-1* shortened the lifespan of the nematode *C. elegans* 148, whereas loss of H3K4me3 is prolonging it 155, further supporting the context-dependency of histone modifications.

### Histone acetylation

As outlined above, normal aging of yeast cells is accompanied by a profound loss of histone proteins 91. The removal of histones from DNA and the incorporation of histones onto DNA are mediated by so-called histone chaperones. A highly conserved central chaperone of histones H3 and H4 is Antisilencing function 1 (Asf1), which is required for proper regulation of gene expression, acetylation of H3 on K56 (H3K56ac; Table 1), and the maintenance of genomic integrity 149,150,151. Deletion of *asf1* results in a very short CLS and a median RLS of only about 7 generations in comparison to the median life span of about 27 generations for wild-type yeast and of 15 generations for *sir2* mutants 91. Yeast lacking both *asf1* and *sir2* are extremely short lived with a median lifespan of 4 to 5 generations, which demonstrates that Asf1 and Sir2 are acting independently to promote longevity. 

The role of Asf1 in determining a normal lifespan is mediated via acetylation of H3K56, which is mediated by the histone acetyltransferase (HAT) Rtt109 (Table 1) 152. In lifespan regulation Asf1 and Rtt109 act together and a H3K56Q mutant, which mimics acetylation, has a greatly shortened lifespan. Similarly, yeast strains lacking the two redundant histone deacetylases Hst3 and Hst4 (Table 1), which leads to elevated levels of H3K56ac 153,154, die early, indicating that H3K56ac levels need to be tightly regulated 91. H3K56ac levels appear to administer histone gene expression, which is repressed by the histone information regulator (HIR) complex 155,156. Consequently, overexpression of the four core histones extends the median lifespan of *asf1* mutants by 65% and inactivation of any component of the Hir complex (Hir1, Hir2, Hir3, Hir4, respectively) extends the median RLS of yeast cells by 25% – 35%. On the contrary, overexpression of *HIR1* suppressed mRNA-instability induced apoptosis of yeast cells lacking a functional *LSM4*, a component of the U6 snRNA complex, during chronological aging, coinciding with a prolonged CLS 157. How alterations in Hir proteins lead to lifespan extension on a mechanistic level remains to be seen, but it appears to be via a pathway that is independent of known lifespan regulators, such as Sir2 and the TOR pathway 91. Interestingly, however, the loss of core histones is also directly correlated to aging of primary human fibroblasts 158.

Dying ∆*asf1* cells not only show marks of apoptosis, but also of necrosis and they accumulate a multitude of autophagic bodies 159. Autophagy (also called macroautophagy) is an evolutionary conserved process important for human health by which cytoplasmic contents, such as damaged organelles or aggregated proteins, are degraded by the lysosome/vacuole. Autophagy is primarily a pro-survival process, but it can also contribute to cell death, and it is becoming increasingly clear that the transcriptional regulation of autophagy-related genes is partially controlled by histone modifications and that this serves as a key determinant of survival versus death decision in autophagic cells 160. Key in this context appears to be the loss of H3 and/or H4 acetylation, in yeast as well as in higher eukaryotes. 

First evidence therefor came from studies on chronologically aged cells: chronologically aging and dying yeast cells show a decline in the levels of polyamines, a typical hallmark of aging across evolution 161,162,163. Treating wild-type yeast cells with the exogenous polyamide spermidine extended the CLS due to deacetylation of histone H3 at K9, K14, and K18 through the inhibition of HATs, which coincided with suppression of oxidative stress and necrotic cell death 161. The altered acetylation status of histone H3 led to a significant up-regulation of autophagy-related genes and consequently autophagy activation, not only in aging yeast, but also in *Drosophila*, *C. elegans*, and human cells 161. Hyperacetylation of H3 at K14 and K18, but not K9, due to defects in acetate metabolism on the contrary led to a dramatically reduced CLS of yeast cells, which appeared to be due to the inability of the yeast mutants to induce autophagy 164,165. This negative effect of hyperacteylated H3 on cellular aging appears conserved across species 164,165.

The critical linkage between histone modifications, the transcriptional regulation of autophagy-related genes and cell death is further supported by the observation that a decrease in H4K16ac due to the down-regulation of the HAT hMOF emerged from the induction of autophagy in distinct mammalian cell lines 166. Similarly, the expression of Sas2, the yeast homolog of hMOF, and H4K16ac levels were found repressed upon autophagy induction in yeast 160,166. The inhibition of H4K16 deacetylation in mammalian cells did not inhibit autophagy, but increased the autophagic flux, whereas reduced H4K16ac was accompanied by the down-regulation of a large number of autophagy-related genes 160,166. Alongside with the reduction of H4K16ac also H3K4me3 is decreased upon autophagy induction in a wide variety of cells from yeast to higher eukaryotes, which may contribute to a general transcriptional inhibition to save energy during prolonged starvation 160. Antagonizing H4K16 deacetylation by overexpressing of hMOF or by inhibiting the HDAC SIRT1, which has H4K16 as its primary histone target, resulted in increased cell death upon autophagy-induction 160,166. In a screen using a library of histone H3-H4 yeast mutants, acetylation of H3K56 was further found reduced in cells treated with the TOR-inhibitor rapamycin, which was proposed to result from the TOR-dependent repression of the H3K56 HDACs Hst3 and Hst4 167. How this relates to programmed cell death remains to be seen.

## POTENTIAL DOWNSTREAM MECHANISMS OF EPIGENETIC REGULATION DURING CELL DEATH

**Figure 2:**
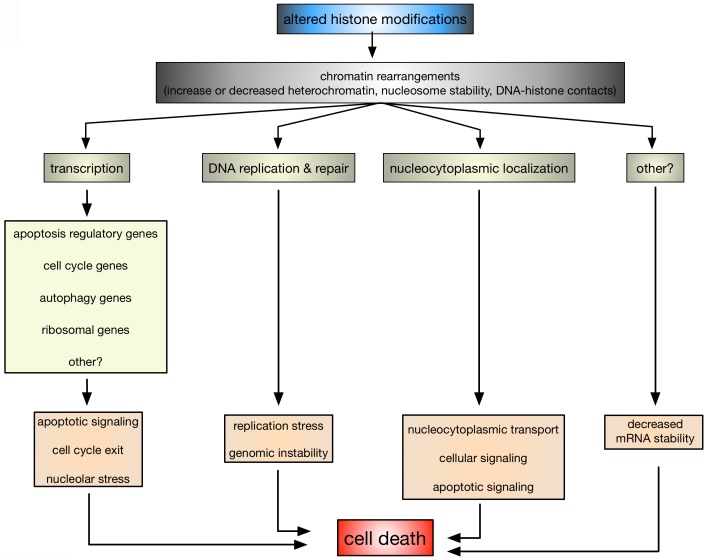
FIGURE 2: Schematic presentation as to how altered histone modifications might promote cell death. Changes in histone modifications will lead to structural rearrangements in the chromatin, which in turn will affect processes, such as transcription, DNA replication and repair, nucleocytoplasmic localization of proteins. Altered transcription may change the expression of regulatory apoptotic factors, cell cycle, autophagy, ribosomal and other vital genes, which in turn will affect apoptotic signalling, cell cycle progression and/or ribosome biogenesis, which may lead to cell death. Replication stress, genomic and mRNA instability, defects in nucleocytoplasmic transport as well as other vital signalling pathways may also lead to cell death as a consequence of altered histone marks.

While it is without any doubt that histone modifications are critical regulators of cell survival and death, it is only poorly understood how they and the coinciding chromatin rearrangements do so on a mechanistic level. The most obvious process influenced by alterations in histone modifications is transcription (Fig. 2). 

In this context it has been shown that altered H2B ubiquitination in ∆*bre1* and ∆*ubp10* cells led to the enhanced expression of *YCA1*, likely due to the inappropriate localization of silencing complexes 34,72,73,76. H2B ubiquitination also plays a role in p53-mediated apoptosis in human cells, although rather indirectly: knockdown of the EHF transcription factor induced p53-dependent apoptosis, whereas its overexpression is required for the survival of p53-positive colon cancer cells 168. EHF directly activates the transcription of RUVBL1, an ATPase associated with chromatin-remodeling complexes. RUVBL1 represses transcription of p53 and its target genes by binding to the p53 promoter, as well as by interfering with RNF20/hBRE1-mediated H2B monoubiquitination and by promoting trimethylation of H3 at K9 (H3K9me3), a transcriptional silencing mark 168. In leukaemia cells, it was furthermore shown that inhibition of DOT1L, the sole human homolog of yeast Dot1, and H3K79 methylation increased apoptosis due to down-regulation of anti-apoptotic BCL2L1 169. Yeast cells lacking *RTT109* or *ASF1* and consequently H3K56 acetylation are characterized by the repression of cell cycle genes and an accumulation of cells with in G2/M phase of the cell cycle 91, whereas H4K16 acetylation is, across species, important for the transcriptional regulation of autophagy-related genes and the subsequent survival versus death response 160,166. Also, ribosomal DNA silencing is affected by changes in H4K16ac 77,84,86,87,91, which may lead to nucleolar stress and an apoptotic response 170. Together these data indicate that altered histone modifications can directly influence apoptosis due to deregulated transcription of key apoptosis regulatory proteins and indirectly due to deregulation of, for example, cell cycle, autophagy, and ribosomal genes.

Impaired DNA DSB repair and replication defects are other nuclear processes that are influenced by histone modifications and might link them to apoptosis (Fig. 2). For example, H2B ubiquitination is required for Rad9-mediated checkpoint activation after DNA damage and Rad51-dependent DNA repair in yeast 63,64 and similarly H3K4 and H3K79 methylation have been implicated in DNA repair as well as DNA replication and recombination 171,172. Indeed yeast cells lacking *set1* and H3K4me have impaired DSB repair and are accumulating mutations 33, as well as replication defects 118, but it remains to be seen if this is related to the increase in apoptotic death of these cells.

Another, rather unexpected regulatory mechanism that is influenced by histone modifications appears to be altered nucleocytoplasmic localization (Fig. 2). In HeLa cells it was shown that cytochrome *c*, released from mitochondria upon apoptosis induction, is translocating into the nucleus, where it specifically binds acetylated H2A 173. Nuclear, H2Aac-bound cytochrome *c* is causing chromatin condensation and further potentiating apoptosis 173. The ING (Inhibitor of Growth) family of tumor suppressors acts as readers and writers of histone modifications and ING1 is a reader of H3K4me3 139. ING1 is a nuclear and nucleolar protein, where it exhibits apoptotic functions, and it translocates to mitochondria of primary human fibroblasts and epithelial cell lines in response to apoptosis stimuli 174. ING1 harbors a BH3-like domain due to which it can bind pro-apoptotic Bax and promote mitochondrial membrane permeability 174. In mammalian cells it was further shown that H2BS14ph is directly contributing to the inactivation of survival pathways, including NF-kB, due to immobilizing the RanGEF RCC1 on chromatin, thereby reducing nuclear RanGTP levels and inactivating nucleocytoplasmic transport 11. 

## CONCLUSION

Apoptotic cell death is accompanied by pronounced structural rearrangements within the cell, including chromatin architecture. Accumulating evidence points towards an epigenetic regulation of the chromatin remodelling events. A key player in this context appears to be monoubiquitination of histone H2B: it shelters yeast cells against intrinsic and extrinsic death stimuli. H2B monoubiquitination is prerequisite for methylation of histone H3K4 and H3K79, respectively, and H3K4 methylation appears as another vitally essential histone mark, whereas the role of H3K79 methylation is less clear. Importantly, the same histone modifications seemingly decide on the fate of higher eukaryotic cells, although context- and tissue-specific variances contribute to and complicate the particular decision. Nevertheless, these studies show that beyond the conservation of the apoptotic core machinery in yeast, also intrinsic triggers of cell death are conserved. Future studies are needed to further dissect the death code of yeast cells and most importantly to further identify and characterize the up- and downstream players that transmit the signals to and from the nucleus.
